# Sensors and Sensing Technologies for Indoor Positioning and Indoor Navigation

**DOI:** 10.3390/s20205924

**Published:** 2020-10-20

**Authors:** Francesco Potortì, Filippo Palumbo, Antonino Crivello

**Affiliations:** Institute of Information Science and Technologies (ISTI), National Research Council (CNR), 56124 Pisa, Italy

**Keywords:** indoor positioning, indoor navigation, location-based services

## Abstract

The last 10 years have seen enormous technical progress in the field of indoor positioning and indoor navigation; yet, in contrast with outdoor well-established GNSS solutions, no technology exists that is cheap and accurate enough for the general market. The potential applications of indoor localization are all-encompassing, from home to wide public areas, from IoT and personal devices to surveillance and crowd behavior applications, and from casual use to mission-critical systems. This special issue is focused on the recent developments within the sensors and sensing technologies for indoor positioning and indoor navigation networks domain. The papers included in this special issue provide useful insights to the implementation, modelling, and integration of novel technologies and applications, including location-based services, indoor maps and 3D building models, human motion monitoring, robotics and UAV, self-contained sensors, wearable and multi-sensor systems, privacy and security for indoor localization systems.

## 1. Introduction

The last 10 years have seen enormous technical progress in the field of indoor positioning and indoor navigation; yet, in contrast with outdoor well-established GNSS solutions, no technology exists that is cheap and accurate enough for the general market.

The potential applications of indoor localization are all-encompassing, from home to wide public areas, from IoT and personal devices to surveillance and crowd behavior applications, and from casual use to mission-critical systems.

What researchers and industry are aiming at is something similar to GSNN which works indoors: a low-cost kind of chip that will become standard on smartphones, robots, all types of traceable equipment, and which works in all environments that are equipped with an infrastructure that is no more expensive than WiFi and which is flexible enough to be adaptable to very different performance requirement, from shop-level accuracy in a mall to decimeter-level accuracy and high precision and reliability in industrial environment, from hour-periodic updates for equipment tracing to sub-second responsiveness for sports, from highly connected, well supervised environments such as an airport to unconnected, highly critical environment such as a mine.

We are still very far from such a solution, and it is not obvious at all that such a single solution will ever be available, but the range of approaches to universal indoor localization is still widening after twenty years of research.

Besides regular submissions, this special issue welcomed selected papers from the 10th edition of the International Conference on Indoor Positioning and Indoor Navigation (IPIN 2019) (http://ipin-conference.org/2019/) and the sixth IPIN Competition [1] as extended versions. As a result, it gives an interesting insight on the many diverse solutions that are being laid out in the sensors landscape.

## 2. Contributions

In this special issue, we collected 23 insightful contributions focusing on the various aspects of the indoor positioning and indoor navigation theme. Furthermore, a meta-review paper has also been submitted and accepted that focuses on technologies, techniques, and approaches used in Indoor Positioning Systems (IPSs). The meta-review allows the reader to inspect the current state in IPS at a glance and serve as a guide for the reader to easily find further details on each technology used in IPS [2].

Among the various technologies and techniques exploited by the proposed systems, we find the Inertial Measurement Units (IMUs) used for Pedestrian Dead Reckoning (PDR) and tracking. In [3], the authors show a PDR system which uses IMUs only to estimate positions by combining velocity and orientation. The authors claim interesting advances in this field through a combined implementation of Convolutional Neural Network (CNN), bidirectional recurrent neural network (BLSTM) and a linear Kalman Filter (KF). The system shows better performances in terms of velocity and traveled distance estimation if compared with the state of the art. IMU based systems generally suffer from unbounded errors in the position and orientation estimates. To overcome these issues, in [4], the authors propose applying prior knowledge instead of using exteroceptive sensors for track cycling. In their paper, the authors effectively show that the use of prior knowledge can yield full observability of the position and orientation. Extending their previous studies, this paper analyzes the observability and improves the pose estimation in the experiment they have conducted. The improved estimator is presented and evaluated on a dataset with three 60-round trials (10 km each). The zero-velocity update (ZUPT)-aided extended Kalman filter (EKF) is commonly used in the traditional inertial navigation system (INS)-based foot-mounted PDR systems, which can effectively suppress the error growth of the inertial-based pedestrian navigation systems. However, in the realistic test, the system still often suffers from drift, which is commonly caused by two reasons: failed detection of the stationary phase in the dynamic pedestrian gait and heading drift, which is a poorly observable variable of the ZUPT method. In [5], in order to improve the initial heading alignment accuracy, a novel method to calibrate the PDR system’s initial absolute heading is proposed which is based on the geometric method. Furthermore, for the problem of failed detection of the stationary phase in the dynamic pedestrian gait, a novel stationary phase detection method is proposed, which is based on foot motion periodicity rather than the threshold comparison principle in the traditional method.

From the submitted contributions we can see that machine learning is widely used to implement tracking systems. In [6], the authors focus on target tracking and the authors propose an interesting approach based on Support Vector Machine (SVM) and KF in order to reduce the bias introduced by low stability and de-calibration effects. The key impact in this work is a dynamic choice of the threshold value during the innovation update phase in the traditional KF-based estimation. Finally, the authors demonstrate the robustness of their own algorithm in a wide set of simulations and in real-world scenarios considering several paths and by including turning regions. In fact, tracking in turning regions constitutes a major challenge in target tracking. In these experiments, the system proposed achieved an optimal tracking performance in most cases. Machine Learning (ML) is also used to implement fingerprinting methods, as seen in [7]. The paper proposed a way to overcome the problem of changes in indoor environments when using Received Signal Strength Indicator (RSSI) fingerprints that can lead to low performance using a a Hybrid Wireless fingerprint (HW-fingerprint) based on a Convolutional Neural Network (CNN). The fingerprint method has been widely adopted in Wi-Fi indoor positioning because of its advantage in non-line-of-sight channels between Access Points (APs) and mobile users. However, the RSS during the fingerprint positioning process generally varies due to the dissimilar hardware configurations of heterogeneous smartphones. This difference may degrade the accuracy of fingerprint matching between fingerprint and test data. Thus, in [8] the authors put forward a fingerprint method based on Grey Relational Analysis (GRA) to approach the challenge of heterogeneous smartphones and to improve positioning accuracy.

Machine learning-based indoor localization used to suffer from the collection, construction, and maintenance of labeled training databases for practical implementation. Semi-supervised learning methods have been developed as efficient indoor localization methods to reduce use of labeled training data. To boost the efficiency and the accuracy of indoor localization, the work presented in [9] proposes a new time-series semi-supervised learning algorithm. The key aspect of the developed method, which distinguishes it from conventional semi-supervised algorithms, is the use of unlabeled data. The learning algorithm finds spatio-temporal relationships in the unlabeled data, and pseudolabels are generated to compensate for the lack of labeled training data. In the next step, another balancing-optimization learning algorithm learns a positioning model. The proposed method is evaluated for estimating the location of a smartphone user by using a Wi-Fi RSSI measurement.

Wi-Fi and models employing CNN and Gaussian Process Regression (GPR) based on RSSI fingerprinting data are also used in [10]. In the proposed scheme, the CNN model is trained by a training dataset. The trained model adapts to complex scenes with multipath effects or many access points. More specifically, the pre-processing algorithm makes the RSSI vector which is formed by considerable RSSI values from different APs readable by the CNN algorithm. The trained CNN model improves the positioning performance by taking a series of RSSI vectors into account and extracting local features. In this design, however, the performance is to be further improved by applying the GPR algorithm to adjust the coordinates of target points and offset the over-fitting problem of CNN.

As we can see, most researchers focus on the improvement of online positioning algorithms using RSSI values. In [11] instead, the authors also use the Media Access Control (MAC) addresses received from the WLAN. They attempt to integrate MAC addresses and RSSI values simultaneously in order to realize indoor localization within multi-story buildings. A novel approach to indoor positioning within multi-story buildings is presented, which includes two steps: firstly, to identify the floor using the difference of received MAC addresses in different floors; secondly, to implement further localization on the same floor. Meanwhile, clustering operation using MAC addresses as the clustering index is introduced in the online positioning phase to improve the efficiency and accuracy of indoor positioning.

A big role in the usage of wireless signals from device pervasively available in indoor environments is played by the features offered by the Wi-Fi standard, like Fine Time Measurements (FTM) of the Round Trip Time (RTT) and Channel State Information (CSI). In [12], the authors investigate how improve the accuracy in systems based on FTM/RTT. In particular, starting from the assumption that in such systems the error in position depends on several factors, including the bandwidth of the RF signal, delay of the signal due to the high relative permittivity of construction materials, and the geometry-dependent “noise gain” of position determination, the authors introduce the so called “frequency diversity”, a method for doubling the accuracy of indoor position determination using weighted averages measurements with uncorrelated errors obtained in different channels. The properties of this method are verified experimentally with a range of responders. Finally, different ways of using the distance measurements to determine indoor position are discussed and the Bayesian grid update method shown to be more useful than others, given the non-Gaussian nature of the measurement errors. For what concerns CSI, its innate variation in the signals may lead to an increase in fingerprint noise and inaccurate data classification. In [13] an indoor localization algorithm is presented, based on Density-Based Spatial Clustering of Applications with Noise (DBSCAN), Endpoints-Clipping (EC) CSI amplitude, and Support Vector Machine (EC-SVM). In the offline phase, the CSI amplitude information collected through the three channels is combined and clipped using the EC, and then a fingerprint database is obtained. In the online phase, the SVM is used to train the data in the fingerprint database, and the corresponding relationship is found with the CSI data collected in real time to perform matching and positioning. The experimental results show that the positioning accuracy of the EC-SVM algorithm is superior to the state-of-art indoor CSI-based localization technique.

In the indoor positioning scenario, passive position detection has gained widespread attention. CSI, as it can provide more detailed and fine-grained information, has been followed by researchers to address this goal. In [14], a CSI-based passive indoor positioning method was proposed, Wavelet Domain Denoising (WDD) was adopted to deal with the collected CSI amplitude, and the CSI phase information was unwound and transformed linearly in the offline phase. The post-processed amplitude and phase were taken as fingerprint data to build a fingerprint database, correlating with reference point position information. Results of experimental data analyzed under two different environments show that the present method boasts lower positioning error and higher stability than similar methods and can offer decimeter-level positioning accuracy.

Indoor passive localization is also the focus of the approach described in [15], called eigenspace-based DOA with direct-path recognition (ES-DPR). It is based on a Direction of Arrival (DOA) estimation algorithm with multiple omnidirectional antennas deployed in a uniform linear array (ULA). To address the multipath propagation interference problem in the indoor environments, the authors utilize the azimuth and RSS estimation results, which are calculated by using the eigenspace-based DOA (ES-DOA) algorithm. A direct-path bearing recognition algorithm is introduced to identify the real DOA of the signal source in different indoor environments, by combining the azimuth and RSS estimation with ensemble learning methods.

In addition, Bluetooth Low Energy (BLE) is commonly used as wireless technology to address indoor positioning. The authors in [16] face the challenges related to RSSI fluctuations and the effect of human body shadowing when using BLE. In order to mitigate these effects, the paper proposes a dynamic Artificial Intelligence (AI) model that uses the three different BLE advertising channels to detect human body shadowing and compensate the RSSI values accordingly. Experiments conduct in real-world scenario, in particular an office environment, show the efficacy of the AI model to detect and compensate RSSI values for a dynamic blockage caused by a human body.

The authors in [17] report and evaluate two approaches of collaborative positioning by combining Wi-Fi and Bluetooth data in order to improve the overall performances in tracking a device. These two approaches are distinguished in non-temporal and temporal. In the non-temporal approach, the author’s model establishes an error observation function in a specific interval of the Bluetooth and Wi-Fi output and, then, try to reduce the positioning error by minimizing the error function. The temporal approach employs an extended error model that takes into account the time component between users’ movements. The authors have shown the system’s performances into several indoor environment whit multi-user. Results show that the collaborative positioning based on the Wi-Fi and Bluetooth data in a multi-user context improves the positioning accuracy for specific scenario.

Wireless Sensor Networks (WSNs) integrating sensor technologies, embedded computing, and networking/communication capabilities have also become research hotspots for indoor localization applications. The positioning errors of wireless sensor networks are mainly caused by the non-line of sight (NLOS) propagation, occurring in complicated channel environments such as the indoor conditions. To this end, in [18], a localization method using a robust extended Kalman filter and track-quality-based (REKF-TQ) fusion algorithm is proposed to mitigate the effect of NLOS errors.

From the technological point of view, besides IMUs, RSSIs, Wi-Fi and BLE features, a wide range of navigation applications make use of cameras. In [19], Simultaneous Localization and Mapping (SLAM) application is presented that can be used in autonomous driving and augmented reality. The authors have developed an improved monocular visual SLAM system by using omnidirectional cameras.

Augmented Reality is also used to provide navigation systems. In [20] the authors present a vision-based navigation system which run entirely on a commercial smartphone. The authors propose an interesting approach using Augmented Reality (AR) and mixed reality tools. The AR tools are used as visual pedometry (scaled optical flow) sensors. Combining a particle filter with a novel mapping solution the system is able to perform accurate positioning evaluations for various indoor applications. Through several simulations and experiments in real-world scenarios, the authors show remarkable accuracy performances which ranges from 1 m to 2 m in most of the scenarios considered.

Other technologies used in the presented systems are ultrasounds and Visible Light Communication (VLC). The authors in [21] reports interesting advances in 3D scenarios by proposing an ultrasound-based approach. In fact, the authors propose an ultrasonic local positioning system (ULPS), based on a set of three asynchronous ultrasonic beacon units and on a 3D mobile receiver prototype. The final position is obtained by merging the partial results from each unit, implementing a minimum likelihood estimation (MLE) fusion algorithm. The approach has been characterized, and experimentally verified, trying to maximize the coverage zone, at least for typical sizes in most common public rooms and halls. Their proposal has achieved a positioning accuracy below decimeters for 90% of the cases in the zone where the three ultrasonic beacon units are available.

The authors in [22] investigate the visible light communication (VLC) techniques applied to industrial scenarios. In order to avoid flickering effects, the authors observe that the transmission frequency is usually much higher than the sampling frequency of ordinary cameras. This aspect led to undersampling issues. In their paper, a potential problem of undersampled protocols is highlighted. Furthermore, the authors report an error analysis as a function of protocol parameters and various error sources. Based on the results, a robust-undersampled phase-shift on-off keying (UPSOOK) protocol is introduced, which guarantees the correct operation even in the presence of clock inaccuracies, as well as other error sources such as sensor noise, jitter and camera saturation. The properties of the proposed robust-UPSOOK protocol are demonstrated using simulations and measurements. In the same context of the Visible Light Positioning (VLP), in which light emitting diode (LED) luminaries are used as positioning beacons, the authors in [23] exploit the quadrature angular diversity aperture (QADA) as a new receiver designed specifically for VLP systems using angle-of-arrival estimation. Starting from previous QADA research that has focused only on positioning and assumed error-free communication, this paper investigates, via simulations and experiment, the actual communication characteristics of a VLP system that uses a QADA receiver. The authors show that reliable communication is assured in typical operating scenarios, proving that communication will not be a limiting factor when using QADA in VLP systems. The authors in [24] report an interesting analysis about which techniques give better results to lay the foundations for the development of a Visible Light Positioning system (VLP). Working only with a receiver, it is analyzed what the result of determining the position of different emitters is when they emit simultaneously and without any synchronism. As a result of their research, the advantages and disadvantages using different multiple-access determination techniques are determined. Their work, through simulations and empirical tests concludes that IPS systems based on optical signals and position sensitive device can achieve very high measurement accuracy.

## References

[1] Potortì F., Crivello A., Palumbo F. (2019). The EvAAL evaluation framework and the IPIN competitions. Geographical and Fingerprinting Data to Create Systems for Indoor Positioning and Indoor/Outdoor Navigation.

[2] Mendoza-Silva G.M., Torres-Sospedra J., Huerta J. (2019). A meta-review of indoor positioning systems. Sensors.

[3] Feigl T., Kram S., Woller P., Siddiqui R.H., Philippsen M., Mutschler C. (2020). RNN-aided human velocity estimation from a single IMU. Sensors.

[4] Koller T.L., Frese U. (2020). State Observability through Prior Knowledge: Analysis of the Height Map Prior for Track Cycling. Sensors.

[5] Zhang W., Wei D., Yuan H. (2019). Novel drift reduction methods in foot-mounted PDR System. Sensors.

[6] Wang X., Liu X., Wang Z., Li R., Wu Y. (2020). SVM+ KF Target Tracking Strategy Using the Signal Strength in Wireless Sensor Networks. Sensors.

[7] Liu Z., Dai B., Wan X., Li X. (2019). Hybrid Wireless Fingerprint Indoor Localization Method Based on a Convolutional Neural Network. Sensors.

[8] Zhang S., Guo J., Luo N., Zhang D., Wang W., Wang L. (2019). A Calibration-Free Method Based on Grey Relational Analysis for Heterogeneous Smartphones in Fingerprint-Based Indoor Positioning. Sensors.

[9] Yoo J. (2019). Time-Series Laplacian Semi-Supervised Learning for Indoor Localization. Sensors.

[10] Zhang G., Wang P., Chen H., Zhang L. (2019). Wireless Indoor Localization Using Convolutional Neural Network and Gaussian Process Regression. Sensors.

[11] Han L., Jiang L., Kong Q., Wang J., Zhang A., Song S. (2019). Indoor Localization within Multi-Story Buildings Using MAC and RSSI Fingerprint Vectors. Sensors.

[12] Horn B.K. (2020). Doubling the Accuracy of Indoor Positioning: Frequency Diversity. Sensors.

[13] Hao Z., Yan Y., Dang X., Shao C. (2019). Endpoints-clipping CSI amplitude for SVM-based indoor localization. Sensors.

[14] Dang X., Tang X., Hao Z., Liu Y. (2019). A device-free indoor localization method using CSI with Wi-Fi signals. Sensors.

[15] Chen Z., Wang J. (2019). ES-DPR: A DOA-based method for passive localization in indoor environments. Sensors.

[16] Naghdi S., O’Keefe K. (2020). Detecting and Correcting for Human Obstacles in BLE Trilateration Using Artificial Intelligence. Sensors.

[17] Ta V.C., Dao T.K., Vaufreydaz D., Castelli E. (2020). Collaborative Smartphone-Based User Positioning in a Multiple-User Context Using Wireless Technologies. Sensors.

[18] Wang Y., Jie H., Cheng L. (2019). A Fusion Localization Method based on a Robust Extended Kalman Filter and Track-Quality for Wireless Sensor Networks. Sensors.

[19] Liu S., Guo P., Feng L., Yang A. (2019). Accurate and Robust Monocular SLAM with Omnidirectional Cameras. Sensors.

[20] Landau Y., Ben-Moshe B. (2020). STEPS: An Indoor Navigation Framework for Mobile Devices. Sensors.

[21] Mannay K., Ureña J., Hernández Á., Machhout M., Aguili T. (2020). Characterization of an Ultrasonic Local Positioning System for 3D Measurements. Sensors.

[22] Rátosi M., Simon G. (2020). Robust VLC Beacon Identification for Indoor Camera-Based Localization Systems. Sensors.

[23] Mohammed M., He C., Cincotta S., Neild A., Armstrong J. (2020). Communication Aspects of Visible Light Positioning (VLP) Systems Using a Quadrature Angular Diversity Aperture (QADA) Receiver. Sensors.

[24] De-La-Llana-Calvo Á., Lázaro-Galilea J.L., Gardel-Vicente A., Rodríguez-Navarro D., Rubiano-Muriel B., Bravo-Muñoz I. (2020). Analysis of Multiple-Access Discrimination Techniques for the Development of a PSD-Based VLP System. Sensors.

